# Clinical Assessment of Ventricular Wall Stress in Understanding Compensatory Hypertrophic Response and Maladaptive Ventricular Remodeling

**DOI:** 10.3390/jcdd8100122

**Published:** 2021-09-29

**Authors:** Takeshi Tsuda

**Affiliations:** 1 Nemours Cardiac Center, Nemours Children’s Hospital Delaware, 1600 Rockland Rd, Wilmington, DE 19803, USA; ttsuda@nemours.org; Tel.: +1-(302)-651-6677; Fax: +1-(302)-651-6601; 2 Department of Pediatrics, Sidney Kimmel Medical College at Thomas Jefferson University, 11th and Walnut Street, Philadelphia, PA 19107, USA

**Keywords:** remodeling, heart failure, hypertrophy, maladaptive transition, echocardiogram, magnetic resonance imaging

## Abstract

Ventricular wall stress (WS) is an important hemodynamic parameter to represent myocardial oxygen demand and ventricular workload. The normalization of WS is regarded as a physiological feedback signal that regulates the rate and extent of ventricular hypertrophy to maintain myocardial homeostasis. Although hypertrophy is an adaptive response to increased biomechanical stress, persistent hypertrophic stimulation forces the stressed myocardium into a progressive maladaptive process called ventricular remodeling, consisting of ventricular dilatation and dysfunction in conjunction with the development of myocyte hypertrophy, apoptosis, and fibrosis. The critical determinant of this pathological transition is not fully understood, but an energetic mismatch due to uncontrolled WS is thought to be a central mechanism. Despite extensive basic investigations conducted to understand the complex signaling pathways involved in this maladaptive process, clinical diagnostic studies that translate these molecular and cellular changes are relatively limited. Echocardiographic assessment with or without direct measurement of left ventricular pressure used to be a mainstay in estimating ventricular WS in clinical medicine, but in recent years more and more noninvasive applications with magnetic resonance imaging have been studied. In this review article, basic clinical applications of WS assessment are discussed to help understand the progression of ventricular remodeling.

## 1. Ventricular Remodeling as a Dynamic, Pathological Process Leading to Symptomatic Heart Failure

Heart failure is a progressive lethal disease that is a major cause of morbidity and mortality in the modern world [1]. Ventricular myocardium possesses a dynamic compensatory capacity to maintain necessary oxygen delivery to the peripheral tissues. Myocardial hypertrophy initially develops as a protective and adaptive growth mechanism in response to increased ventricular workload and/or a decrease in intrinsic myocardial function, but the disruption of this regulatory adaptational process can result in maladaptive remodeling responsible for progressive hemodynamic deterioration [2,3,4]. It is thus essential to elucidate the underlying regulatory mechanisms that protect the myocardium from developing pathological transition [5,6,7,8,9].

**Figure 1:**
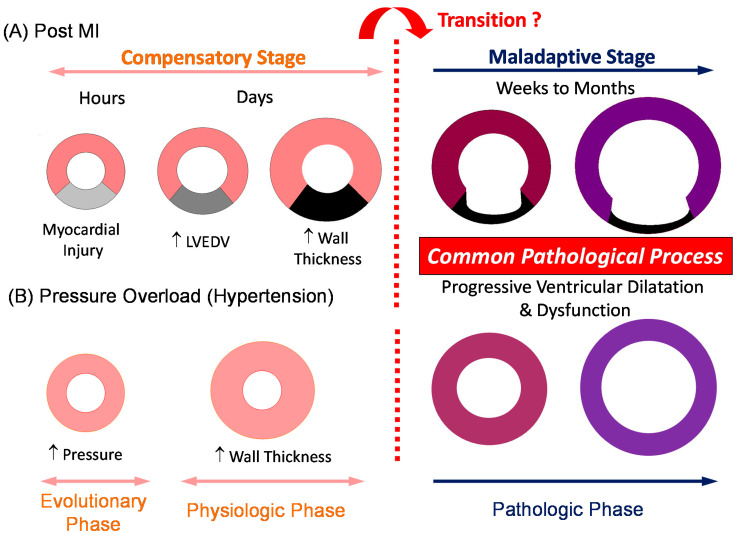
A sequence of ventricular remodeling in post-myocardial infarction (post-MI) (**A**) and persistent pressure overload (**B**) models from compensatory (evolutionary and physiologic) to maladaptive (pathologic) stages. During the compensatory stage, the ventricular myocardium undergoes initial morphological and biochemical changes (compensatory responses) to maintain necessary cardiac output and to regulate myocardial oxygen demand under control. A pathologic transition into an irreversible and progressive stage may occur when the regulatory capacity of myocardial oxygen demand is disrupted. LVEDV = left ventricular end-diastolic volume; MI = myocardial infarction.

Ventricular remodeling refers to a maladaptive pathological process consisting of progressive myocardial hypertrophy, fibrosis, and myocyte cell death in conjunction with ventricular dysfunction and dilatation [2,3,4,5,6,7,8,9,10]. Historically, ventricular remodeling following myocardial infarction, which not only involves ischemic myocardium but also remote nonischemic myocardium, leading to hemodynamic deterioration and symptomatic heart failure, has been studied extensively [11,12,13]. A complex series of transcriptional, signaling, neurohormonal, structural, electrophysiological, and functional events occur within the cardiac myocytes as well as in the extracellular matrix [2,7,8,9]. Weber et al. summarized this transition into three phases in the pressure-overloaded myocardium. In a first evolutionary phase, the myocardium shows initial adaptational responses with preserved pump function and oxygen delivery. The second physiologic phase is characterized by reversible structural and biochemical remodeling within a coordinated balance. This is followed by a pathologic phase that is progressive and irreversible with compromised pump function and oxygen delivery [14]. Thus, the assessment of myocardial deformation is an essential determinant in understanding the progression of ventricular remodeling (Figure 1).

Initial myocardial deformation or persistent stretching of the myocyte activates a complex set of signaling pathways mainly mediated by a stretch sensor integrin, many of which appear to enhance the hypertrophic response either by autocrine/paracrine factors (angiotensin II, interleukin-6 family, insulin growth factor-1, and possibly endothelin-1) or direct activation of the ion channels, Na^+^/H^+^ exchanger, or heterotrimeric G proteins of the Gq and Gi class [15]. Initial ventricular geometric changes, hypertrophy, and chamber dilatation are implemented primarily to maintain cardiac output and to normalize the increased myocardial oxygen demand. However, unresolved myocardial energetic abnormality continues to activate these signaling pathways, resulting in progressive and irreversible ventricular remodeling responsible for congestive heart failure. To understand the progression of ventricular remodeling, it is critical to reliably assess the degree of myocardial stretch or deformation. Wall stress (WS) is an important physiological marker that represents the mechanical and geometric changes in the ventricular myocardium.

## 2. Physiological Significance of Ventricular Wall Stress

Early recognition and assessment of subclinical ventricular geometric changes are essential in identifying the progression of ventricular remodeling. Increased ventricular volume, hypertrophy, decreased ventricular function, and increased systolic WS are common parameters used to measure the overall ventricular performance and severity of the myocardial disease [16,17]. Systolic WS not only represents the ventricular afterload status against which the myocardium has to work to pump out blood, but also elucidates the degree of myocardial deformation reflecting a combination of primary disease processes and secondary compensatory responses [10].

Physiological significance of WS stems from the following: (1) myocardial WS is one of the primary determinants of myocardial oxygen consumption [18,19,20]; (2) normalization of WS is thought to be a physiological feedback signal that regulates the hypertrophic response to minimize an excessive workload on the ventricular myocardium [4]; and (3) interactions between WS, geometric changes, and energetic outcome consist of a fundamental process in understanding ventricular mechanics [21]. When ventricular cavity size enlarges after myocardial infarction or left ventricular (LV) pressure increases with persistent pressure overload (PO), the ventricular myocardium undergoes eccentric or concentric hypertrophy, respectively, to normalize WS in order to minimize the increase in myocardial oxygen demand. As long as the oxygen supply and demand balance in the myocardium is controlled, the ventricular myocardium can maintain an ordinary cardiac output without pathologic transition. Once this balance is jeopardized, the progressive pathologic remodeling proceeds with hemodynamic deterioration and symptomatic congestive heart failure (see Figure 1). However, activation of stretch-induced signaling pathways induces pathological transformation of the myocardial tissue, including myocyte hypertrophy, apoptosis, and extracellular matrix changes (fibrosis), even in subclinical stages when the cardiac output is still preserved [15,22,23]. It is critical to identify this early phase of ventricular remodeling.

## 3. Transition for Compensatory Hypertrophy to Maladaptive Remodeling: Is Compensatory Hypertrophy Always Benign?

Early pioneering work by Grossman et al. proposed a stress-adaptation hypothesis in which increased ventricular wall thickness is induced by an adaptive response based on the law of Laplace [24]. Although conventional WS theory supports the physiological need of hypertrophy that primarily normalizes the increased WS, the development of compensatory hypertrophy in response to increased biomechanical stress (e.g., chronic PO) may not always be an adaptive physiological response [25]. Comprehensive review articles discussing the mechanisms of physiological and pathological cardiac hypertrophy are found elsewhere [9,26]. Gene expressions and signaling pathways noted in physiological hypertrophy and maladaptive remodeling commonly differ (cell growth, cell survival, fatty acid oxidation, and mitochondrial biogenesis vs. inflammation, apoptosis, fibrosis, glycolysis, and fetal isoforms), and some are involved in both physiological and pathological processes [25]. Pathological transition is characterized by further activation of the neurohormonal and cytokine system and consequent microscopic myocardial changes at cellular and tissue levels, including fibrosis and apoptosis [26]. However, the distinction between benign compensatory hypertrophy and pathological maladaptive remodeling is not always clear [27].

Pure physiological or compensatory hypertrophy is seen in physical growth and maturation, athletes (exercise-induced hypertrophy), and during pregnancy. Echocardiographic assessment of LV myocardial mass in children up to age 12 revealed an incremental increase of LV mass with age, independent of sex [28]. Exercise-induced cardiac adaptation, especially eccentric hypertrophy in response to high-level endurance training, is regarded as a benign and favorable response that augments ventricular stroke volume, cardiac output, and aerobic fitness [29,30]. Among elite athletes, incomplete resolution of the LV cavity enlargement was observed in 22%, even after 5 years of the cessation of training, suggesting that permanent myocardial damage can occur as a consequence of prolonged training [31]. The heart undergoes modest eccentric hypertrophy in response to sustained volume overload during the second and third trimester of pregnancy, which reverses spontaneously after delivery [32]. This physiological hypertrophy is induced by volume overload, concomitant myocardial stretch, and changes in sex hormones (estrogen) via multiple hypertrophy-related signaling pathways [33]. Even with these physiological hypertrophies, however, pathological transition can occur. Rarely, patients can develop dilated cardiomyopathy late in the pregnancy or early in the puerperium, designated as peripartum cardiomyopathy, the etiology of which remains largely unknown [34].

**Figure 2:**
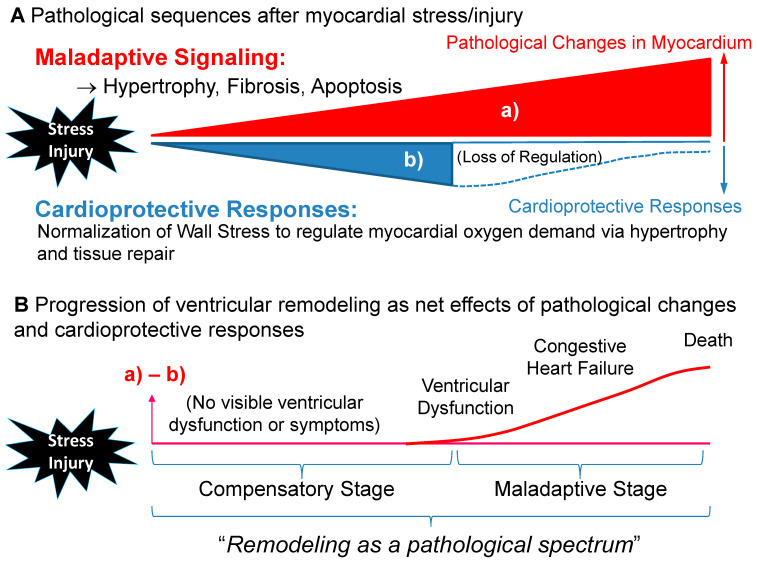
Transition from the compensatory to maladaptive stage in ventricular remodeling. (**A**) After myocardial stress or injury, ventricular myocardium is exposed to both (a) pathological changes and (b) cardioprotective responses. (**B**) Progression of ventricular remodeling may be presented as net effects of (a) pathological changes and (b) cardioprotective responses. Thus, ventricular dysfunction may be masked by initial compensatory cardioprotective responses (hypertrophy) despite activation of the multiple maladaptive signaling pathways induced by persistent abnormal myocardial stretch or biomechanical stretch. The net outcome between maladaptive and cardioprotective responses may determine the overall ventricular dysfunction and systemic perfusion. It is plausible that pathological alteration is already initiated in the early phase and that the maladaptive phase commences when compensatory responses fail to normalize wall stress (Figure 1).

The presence of hypertrophy is widely known as a major risk factor for increased cardiovascular morbidity and mortality in adulthood [35,36,37,38], implying that physiological hypertrophy may not always be a benign adaptation. Two groups challenged the longstanding hypothesis that compensatory hypertrophy acts to maintain normal cardiac function by normalizing WS. Hill et al. demonstrated that the abolition of the hypertrophic response to PO by inhibiting calcineurin with cyclosporin A prevented LV hypertrophy and attenuated LV decompensation [39]. A similar observation was reported by Esposito et al. who proved that the development of hypertrophy and normalization of WS may not be necessary to preserve cardiac function by using genetically engineered mice and their wild type (WT) controls in a PO model [40]. On the contrary, PO-induced hypertrophied WT mice showed progressive deterioration in cardiac function and LV chamber enlargement. These findings suggest that PO-induced hypertrophy is not always required to preserve ventricular performance. A sustained PO is often due to a mixed stimulus that produces both beneficial and adverse maladaptive remodeling simultaneously, by which the WS correction theory does not always apply [27] (Figure 2). Further research is warranted to delineate this complex biological response to PO.

## 4. Clinical Significance of Ventricular Wall Stress 

Quantification of ventricular WS is important as increased WS plays an integral role in understanding the development and progression of LV remodeling. Ventricular WS is estimated according to Laplace’s law, where the heart is modeled by a sphere using the formula below (P: pressure, r: radius, h: wall thickness).

WS = P·r/h

Sandler and Dodge [41] approximated LV as a thin-walled ellipsoid and derived the formula into that shown below.

WS = P·r^2^/h·(2r + h)

Originally, WS was obtained through simultaneous measurement of LV pressure by cardiac catheterization and echocardiographic measurement of LV dimensions [24,42]. Later, systolic blood pressure by cuff measurement was proven to be a reliable surrogate of peak LV pressure, and this enabled us to calculate WS noninvasively with echocardiographic measurement and simultaneous cuff blood pressure measurement [42,43]. Reichek et al. showed a close correlation between invasively measured end-systolic LV pressure and noninvasive cuff systolic pressure (noninvasive P = 1.07·end-systolic P + 0.8 mmHg; r = 0.89) and demonstrated that LV end-systolic WS reliably represents LV afterload, which was negatively correlated with LV systolic function [42]. Douglas et al. reported substantial differences in WS measurement between M-mode meridional, two-dimensional meridional, and two-dimensional circumferential stresses [44]. Greim et al. showed an excellent correlation between end-systolic pressure-area product (obtained by the end-systolic cavity area in a two-dimensional echocardiogram multiplied by systolic arterial pressure) and end-systolic WS and proposed that end-systolic pressure-area product may be used as an alternative reliable method to assess LV WS [45]. In a strict sense, end-systolic WS and peak-systolic WS have different definitions and values [46,47,48]. However, other studies indicate that these two WS are comparable [49,50,51], supporting the validity of using cuff-measured systolic blood pressure measurement as a surrogate of end-systolic pressure to estimate end-systolic WS.

End-systolic WS primarily represents afterload status, but its clinical applications are multifold. Colan et al. validated an excellent inverse relationship between peak-systolic WS and the heart rate-corrected velocity of circumferential fiber shortening (Vcf) in healthy patients, and demonstrated that a slope of the regression line of the two represents contractile reserve independent of preload [52]. Fontanet et al. applied this principle to patients with and without left ventricular hypertrophy (LVH), and revealed that patients with LVH were characterized by a decreased contractile state and increased end-systolic WS at the baseline and a blunted myocardial contractile state in response to dobutamine stimulation. The end-systolic WS-Vcf regression line with dobutamine stimulation illustrates an intrinsic myocardial contractile reserve [49]. This specific relationship has been proven to be sensitive to a pharmacologically-induced inotropic state independent of loading condition in an animal model [53]. The relative wellness of the LV myocardium has been illustrated by normal end-systolic WS. Lamers et al. investigated the end-systolic WS-Vcf relationship in children with sickle cell disease (age 9.4 ± 4.1 years) who were subject to an abnormal loading condition due to chronic anemia, and found that children with sickle cell disease had reduced myocardial contractility, suggesting a masked myocardial abnormality in this population [54].

In 56 adult patients with asymptomatic chronic aortic insufficiency, Greenberg et al. demonstrated a decline in LV ejection fraction with exercise in those who had increased end-systolic WS, consistent with an early manifestation of the deterioration of LV systolic function. The authors postulated that a lack of adequate hypertrophy to normalize WS may have resulted in a loss of the compensatory mechanism, which caused a decline in the ejection fraction during exercise [55]. Haykowsky et al. showed that there was no increase in end-systolic WS through a brief heavy leg-press exercise in healthy trained athletes, but there was a 28% increase in sedentary subjects. Attenuated end-systolic WS in athletes was explained by an acute increase in LV wall thickness accompanied by a decline in the LV cavity dimensions [56]. Myocardial oxygen consumption did not change with resistance exercise in healthy trained individuals. Stable end-systolic WS may indicate a relatively healthy myocardium still within a compensatory phase. 

By using speckle-tracking and other advanced echocardiographic technologies in otherwise asymptomatic middle-aged adults, Chirinos and colleagues studied early and late systolic WS in association with echocardiographic parameters of systolic and diastolic LV function. They demonstrated that early systolic WS is associated with a greater longitudinal systolic function and early diastolic relaxation, whereas late systolic WS was independently associated with early diastolic relaxation and decreased longitudinal systolic function, suggesting the role of time-varying myocardial afterload as a determinant of myocardial relaxation [57]. Regional and global myocardial strain can be quantitatively assessed through the motion of speckles identified on routine two-dimensional images representing myocardial deformation and more specific ventricular dynamics less dependent on the loading conditions [58]. The relationship between global strain obtained by speckle-tracking imaging and WS was investigated by Hurlburt et al., who demonstrated a modest but statistically significant inverse relationship between strain and end-systolic WS (r = −0.29; *p* < 0.05) in 60 healthy adult volunteers [59]. This relationship was supported by another group, suggesting that LV stain is, in part, dependent on afterload, and that myocardial systolic function should be evaluated based on the relationship between strain and WS [60].

## 5. Noninvasive Measurement of Wall Stress as a Biomarker for Ventricular Remodeling

Ventricular WS has been studied in conjunction with the development and progression of ventricular remodeling. In an experimental post-myocardial infarction model, increased WS was closely associated with geometric changes in LV, including volume and infarct area [13,23]. The degree of myocyte apoptosis was shown to be closely correlated with LV systolic WS in patients with severe dilated cardiomyopathy [61]. Rohde et al. showed progressive increase in regional end-systolic WS in experimental post-myocardial infarction that was associated with increased extracellular matrix remodeling indicated by the immune-expression of matrix metalloproteinase-9 protein and macrophage infiltrate in a rat model [62]. In 40 symptomatic patients with aortic stenosis, Vanderheyden et al. reported that the plasma B type natriuretic peptide (BNP) level was correlated with both systolic and diastolic WS, not with systolic function, suggesting that BNP may be an excellent screening tool for LV diastolic dysfunction in patients with pressure overload cardiomyopathy and normal systolic function [63]. A similar finding was noted in patients with chronic heart failure, where the plasma BNP showed a closer correlation with end-diastolic WS than any other hemodynamic parameters including systolic WS [64].

Dong et al. introduced a novel concept of integrated wall stress (IWS), which reflected the entire workload sensed by the LV myocardium. They proposed that IWS is a more responsible physiological marker for total LV afterload than one-point measurement of WS. Unchanged IWS with dobutamine stimulation was noted in normal mice, whereas IWS was significantly increased in post-myocardial infarction mice with dobutamine. There was a good correlation between IWS (equivalent to average WS) and a product of peak systolic WS and heart rate (r = 0.70), postulating that a product of peak systolic WS-heart rate serves as a feasible marker for LV workload [65]. Similarly, Devereux and colleagues demonstrated that a product of LV mass, WS, and heart rate correlated well with myocardial oxygen demand in adult patients with hypertension, and was associated with higher rates of myocardial infarction and cardiovascular mortality [66]. The same principal was also studied in patients with aortic stenosis [67].

A summary of WS measurements using echocardiogram is shown in Table 1. 

## 6. Assessing Global and Regional Ventricular Wall Stress by Magnetic Resonance Imaging

Cardiac magnetic resonance imaging (cMRI) has been applied for the assessment of ventricular WS. Instead of diameter changes in M-mode using echocardiogram, cMRI enables utilization of the area measurement (two-dimensional) or volume measurement (three-dimensional) to calculate WS according to Laplace’s law. Similar to echocardiogram, LV WS via cMRI requires LV pressure measurement either by cardiac catheterization or by noninvasive blood pressure measurement, but MRI can provide a more accurate assessment of the three-dimensional LV geometry superior to the echocardiographic approach. 

Auffermann et al. measured LV systolic WS with cine MRI and simultaneous blood pressure measurement (carotid pulse tracing), and assessed WS in 22 patients with LV volume overload due to aortic and/or mitral insufficiency, with and without myocardial disease, to study the clinical significance of systolic WS in identifying those at risk for a poor prognosis after valve surgery [68]. They demonstrated that LV output and WS rise progressively with increasing LV volume overload and that disproportionally high systolic WS relative to regurgitant volume indicates myopathic changes, suggesting that systolic WS is an important determinant to predict post-surgical outcomes. By combining the MRI measurement and LV pressure obtained by cardiac catheterization, Alter and colleagues showed that the BNP level was correlated well with both end-diastolic and end-systolic WS, indicating that the cellular stretch is a major trigger for BNP release [69,70]. These data suggest that the diagnostic use of BNP should primarily be directed to assess ventricular WS rather than the extent of functional impairment of the LV myocardium. The principle of global LV WS calculation based on Laplace’s law is essentially the same between echocardiography and MRI, but it was found that LV WS obtained by echocardiogram was systematically lower than that obtained by MRI [69]. The same group of investigators examined LV end-systolic and end-diastolic WS and late gadolinium enhancement (LGE) in 300 patients with nonischemic dilated cardiomyopathy with MRI. They showed that increased LV WS and LV mass were associated with the degree of LGE, proposing that capillary leakage by excessive myocardial stretching features pathological remodeling [71]. By calculating end-systolic WS with the LV dimension from MRI, and LV systolic pressure estimated from echocardiographic pressure gradient and systolic blood pressure in patients with severe aortic stenosis, end-systolic WS was shown to be significantly correlated with severity of remodeling, neurohormonal activation, and severity of symptoms [72]. Wall stress is an important determinant of heart failure progression and thus may explain the unfavorable prognostic role of LGE [73].

Genet et al. studied a computational model using MRI and personalized computational cardiac mechanics modeling with the finite-element (FE) method, not Laplace’s law, and proposed that validated regional WS can be used to assess ventricular status in response to heart failure treatment [74]. Computational simulation based on FE analysis with MRI was applied to assess the severity of dilated cardiomyopathy by WS represented by the average stress, calculated as the mean value between the longitudinal fiber stress and the cross-fiber stress, which enabled risk-stratification of dilated cardiomyopathy by noninvasively predicting myocardial stress and pump performance [75]. Wollmuth et al. introduced three-dimensional global end-systolic LV WS determined by MRI and FE assessment, and emphasized that MRI-derived WS measurement provides better accuracy for assessing LV performance with chronic aortic insufficiency than that of echocardiographic assessment utilizing simplified spherical geometric shape assumption [76]. 

Zhong et al. introduced a new concept of pressure-normalized stress (σ/P) as an index of WS (σ: WS, P: pressure) by MRI, and compared σ/P with WS obtained with blood pressure measurement in 40 patients with ischemic cardiomyopathy. They demonstrated that σ/P correlated well with the measured WS in all three different regions (remote, border, and infarct zones) and that surgical ventricular restoration reduced both σ/P and WS and improved systolic function by reducing LV volume [77]. The clinical significance of pressure-normalized WS was also asserted by others as a crucial index of geometric influence on WS [78,79]. They demonstrated that increased LV end-diastolic WS preceded LV hypertrophy in patients with nonischemic dilated cardiomyopathy [78]. This volume-based WS index allowed us to approximate the real WS in the absence of invasive pressure measurements. 

## 7. Conclusions

Wall stress is an important concept to understand for the maladaptive transition from physiological to pathological hypertrophy. End-systolic (or peak systolic) WS represents ventricular afterload and estimates myocardial oxygen consumption that can be reliably obtained noninvasively through echocardiogram and simultaneous cuff blood pressure measurement. In combination with other hemodynamic parameters, systolic WS can provide a reliable assessment of afterload status, myocardial oxygen demand, and development of ventricular remodeling. Although the current approaches of estimating WS in M-mode echocardiography may be limited because of its oversimplification of LV geometry, it still provides certain useful information regarding the disease process and prognosis. New computerized approaches with MRI have enabled us to estimate WS based on regional myocardial deformation and volume assessment. Further involvement of advanced technology is warranted for better delineation of the subclinical failing myocardium.

## Figures and Tables

**Table 1:** Clinical Applications of Systolic Wall Stress (WS) in Echocardiogram.

1. End-systolic WS (1): Ventricular afterload
-Inversely correlated with systolic performance
2. End-systolic WS (2): Myocardial oxygen demand/consumption
-End-systolic WS represents primary determinant of myocardial oxygen demand (Strauer et al. 1977 [20])
3. Noninvasive determination of LV end-systolic WS
-Utilization of cuff systolic blood pressure: Well correlated with invasive LV pressure measurement [42,43]
4. End-systolic WS-Vcf relationship (Colan et al. 1984 [52]; Hoit et al. 1997 [53]; Lamers et al. 2006 [54])
-Vcf/HR is inversely related to end-systolic WS
-A marker for load-dependent myocardial contractile status
5. Integrated WS (Dong et al. 2013 [65])
-Average WS over a unit time
-Stable with dobutamine stimulation in healthy heart
-Good correlation with Peak-systolic WS × HR
6. WS-HR-LV mass product (Devereux et al. 2000 [66]; Gerdts et al. 2019 [67])
-Myocardial oxygen demand is estimated from LV mass × end-systolic WS × HR
-Associated with higher mortality and morbidity in patients with aortic stenosis
7. Early and late systolic WS (Chirinos et al. 2013 [57])
-Early systolic WS: Associated with greater longitudinal systolic function and enhanced early diastolic relaxation
-Late systolic WS: Associated with decreased diastolic relaxation and decreased longitudinal systolic function

HR = heart rate; LV = left ventricular; Vcf = velocity of circumferential fiber shortening; WS = wall stress.
